# Detecting Tonic-Clonic Seizures in Multimodal Biosignal Data From Wearables: Methodology Design and Validation

**DOI:** 10.2196/27674

**Published:** 2021-11-19

**Authors:** Sebastian Böttcher, Elisa Bruno, Nikolay V Manyakov, Nino Epitashvili, Kasper Claes, Martin Glasstetter, Sarah Thorpe, Simon Lees, Matthias Dümpelmann, Kristof Van Laerhoven, Mark P Richardson, Andreas Schulze-Bonhage, The RADAR-CNS Consortium

**Affiliations:** 1 Epilepsy Center Department of Neurosurgery Medical Center - University of Freiburg Freiburg im Breisgau Germany; 2 Ubiquitous Computing Department of Electrical Engineering and Computer Science University of Siegen Siegen Germany; 3 Division of Neuroscience Institute of Psychiatry, Psychology & Neuroscience King's College London London United Kingdom; 4 Data Science Analytics & Insights Janssen Research & Development Beerse Belgium; 5 UCB Pharma Brussels Belgium; 6 The RADAR-CNS Patient Advisory Board King’s College London London United Kingdom; 7 National Institute of Health Research Biomedical Research Centre South London and Maudsley NHS Foundation Trust London United Kingdom; 8 see Acknowledgements

**Keywords:** wearables, epilepsy, seizure detection, multimodal data, mHealth, mobile health, digital health, eHealth

## Abstract

**Background:**

Video electroencephalography recordings, routinely used in epilepsy monitoring units, are the gold standard for monitoring epileptic seizures. However, monitoring is also needed in the day-to-day lives of people with epilepsy, where video electroencephalography is not feasible. Wearables could fill this gap by providing patients with an accurate log of their seizures.

**Objective:**

Although there are already systems available that provide promising results for the detection of tonic-clonic seizures (TCSs), research in this area is often limited to detection from 1 biosignal modality or only during the night when the patient is in bed. The aim of this study is to provide evidence that supervised machine learning can detect TCSs from multimodal data in a new data set during daytime and nighttime.

**Methods:**

An extensive data set of biosignals from a multimodal watch worn by people with epilepsy was recorded during their stay in the epilepsy monitoring unit at 2 European clinical sites. From a larger data set of 243 enrolled participants, those who had data recorded during TCSs were selected, amounting to 10 participants with 21 TCSs. Accelerometry and electrodermal activity recorded by the wearable device were used for analysis, and seizure manifestation was annotated in detail by clinical experts. Ten accelerometry and 3 electrodermal activity features were calculated for sliding windows of variable size across the data. A gradient tree boosting algorithm was used for seizure detection, and the optimal parameter combination was determined in a leave-one-participant-out cross-validation on a training set of 10 seizures from 8 participants. The model was then evaluated on an out-of-sample test set of 11 seizures from the remaining 2 participants. To assess specificity, we additionally analyzed data from up to 29 participants without TCSs during the model evaluation.

**Results:**

In the leave-one-participant-out cross-validation, the model optimized for sensitivity could detect all 10 seizures with a false alarm rate of 0.46 per day in 17.3 days of data. In a test set of 11 out-of-sample TCSs, amounting to 8.3 days of data, the model could detect 10 seizures and produced no false positives. Increasing the test set to include data from 28 more participants without additional TCSs resulted in a false alarm rate of 0.19 per day in 78 days of wearable data.

**Conclusions:**

We show that a gradient tree boosting machine can robustly detect TCSs from multimodal wearable data in an original data set and that even with very limited training data, supervised machine learning can achieve a high sensitivity and low false-positive rate. This methodology may offer a promising way to approach wearable-based nonconvulsive seizure detection.

## Introduction

### Background

Epilepsy is one of the most common chronic neurological diseases, with a reported yearly worldwide incidence of more than 60 per 100,000 individuals [1]. Epilepsy also has a remarkably diverse set of indications, with several different types of symptoms and characteristic seizures of varying severity. Seizures are usually distinguished by their onset in the brain, focal or generalized. They can involve a variety of different combinations of symptoms, including impaired awareness or loss of consciousness; cognitive, emotional, or sensory abnormalities; sudden changes in the autonomic nervous system; or motor manifestations such as spasms, automatisms, or tonic and clonic movements of the limbs [2]. These convulsive seizures, particularly focal to bilateral or generalized tonic-clonic seizures (TCSs), are the most dangerous type of epileptic seizures. They imply loss of consciousness and loss of motor control with considerable risk for physical harm and can transition to life-threatening status epilepticus or sudden unexpected death in epilepsy [3]. For the diagnosis and treatment of epilepsy, clinicians rely on patient self-reporting and structured diaries, counting the number of seizures a patient had in a certain time frame. However, personal diaries filled out by the patients themselves have been proven to be very unreliable, with frequent undercounting because of a lack of awareness of seizures [4,5]. An objective seizure diary is therefore needed to obtain valid data on seizure occurrence, contributing to improved guidance for the treatment of people with epilepsy. Wearable nonelectroencephalography (non-EEG) devices (*wearables*) could provide data for such a diary. They are discreet and unobtrusive, contrary to many wearable EEG devices that are often cumbersome and stigmatizing [6], although some less obtrusive wearable EEG systems are in development [7,8]. Moreover, a robust detection of convulsive seizures with wearables, paired with identification of seizure-related risk factors [9], could be of great clinical importance and provide essential information for the identification of seizure-related sudden unexpected death in epilepsy risk factors.

Although seizure detection with non-EEG wearables is a relatively new field in epilepsy research, there have already been some studies that have demonstrated the viability of this kind of system. To date, most studies have concentrated on a single biosignal modality for training a seizure detection model, with a minority using a multimodal approach [10,11]. In essence, there are 4 main biosignal modalities that are recorded from non-EEG wearables used in epilepsy research: (1) *accelerometry* (ACC)—motion-based activity, (2) *electrodermal activity* (EDA)—changes in electrical properties of the skin, (3) *electrocardiography* (ECG) or *photoplethysmography* (PPG)—heart rate and heart rate variability estimation; and (4) *electromyography* electrical muscle activity. ACC is perhaps the most commonly used in related work because it is easy to integrate into wearable hardware and can provide relevant information, especially on movements during motor seizures. ACC signals have been used in both unimodal [12-14] and multimodal [15-17] seizure detection systems. EDA, also called galvanic skin response, has been used in some studies for seizure detection [16,18], as a large EDA change can occur especially in the postictal phase following TCSs [19]. Another modality that has been used is ECG, and its optical counterpart PPG, which uses light reflection to calculate the heart rate from blood volume changes in an unobtrusive manner. Although there have been some studies using ECG [20-23] or PPG [17,18,23,24] signals for epileptic seizure detection, the considerable movements during convulsive seizures frequently render this signal too noisy for accurate ictal heart rate determination. Finally, electromyography is a self-evident modality for detecting seizures with motor components, identifying ictal muscle contraction, and thus has been used for convulsive seizure detection as well [25-28].

### Objective

In this study, we present an automatic seizure detection system for TCSs using supervised machine learning that is straightforward to implement and reproduce. We evaluated the detection model on a newly recorded data set from a multicenter clinical study with wearable non-EEG devices. Finally, we discuss the detection system, its performance, and its limitations and conclude with an outlook of possible further applications for this detection approach.

## Methods

### Data Set

During the course of the study, between July 2017 and February 2020, we collected wearable device data from 243 patients diagnosed with epilepsy: 70.7% (172/243) of patients were recruited at the epilepsy monitoring unit (EMU) in the Epilepsy Center, Medical Center, University of Freiburg, and 29.2% (71/243) of patients were recruited at the EMU in the neurophysiological department of King’s College Hospital, London. Patients with a diagnosis of epilepsy in the age range of 7 to 80 years were recruited, unless they had vigorous involuntary nonepileptic movements. Consecutive patients were admitted to their respective EMU as part of their standard epilepsy clinical care, for differential diagnosis or for presurgical evaluation, and may have had their antiepileptic medication reduced during the recording. All patients were continuously monitored via a video EEG system during their stay in the EMU. Clinical experts (EB and NE) manually reviewed the video and EEG data for all participants and labeled type, onset, and offset for all seizures. Specifically, they also labeled the onset and termination of every motor manifestation, including the tonic and clonic phases of each seizure. These labels were then used as the ground truth in the training and testing phases of the evaluation. Participants wore a variety of different wearable devices across the 2 sites; however, the only device worn by participants from both sites was a wrist-worn device (Empatica E4, Empatica Inc). The study and recording procedures were further described and discussed in the review by Bruno et al [29]. All recruited patients provided written informed consent, and the study procedures were approved by local ethics committees, the ethics committee at the University of Freiburg (538/16), and the London Fulham Research Ethics Committee (16/LO/2209; Integrated Research Application System project ID216316).

All data recorded at the 2 sites were live streamed from each wearable device to 1 base device per participant, running an Android operating system and a custom-developed app. The data were then transmitted from all base devices to a central server and stored for later analysis. The system was developed by the Remote Assessment of Disease and Relapse-*Central Nervous System* consortium and is available as an open-source project on GitHub [30].

Owing to battery limitations, each participant was assigned 2 devices, between which they changed twice daily to ensure continuous recordings. The wearable device recorded 3-axis ACC at a sample rate of 32 Hz, EDA at 4 Hz, and PPG at 64 Hz, which was processed on the device to a blood volume pulse signal. Participants generally wore the device on the arm that was most involved in motor semiology during seizures, that is, the arm that presented the most significant movements. In the set of 10 participants with TCSs included here, each wore the device on their nondominant hand, except for 2 participants who specified that they were ambidextrous.

### Features

An extensive feature set was created from the ACC and EDA signals, encompassing 141 ACC and 10 EDA features, at sliding window sizes of 2, 10, and 20 seconds for the ACC features, and 5, 10, and 20 minutes for the EDA features. PPG signals were not analyzed because of major ictal movement artifacts. Although artifacts in PPG data can still convey information, in that the presence of noise itself can be information, we chose to omit it here in favor of focusing on the other 2 biosignals, because the information of PPG motion artifacts is naturally included in the ACC signal as well. The ACC features included a variety of different time and frequency domain features. The EDA features represented the skin conductance level (SCL), that is, tonic low-frequency EDA changes, and skin conductance response rate (SCRR), that is, phasic or higher-frequency EDA changes, calculated against a baseline.

**Figure 1:**
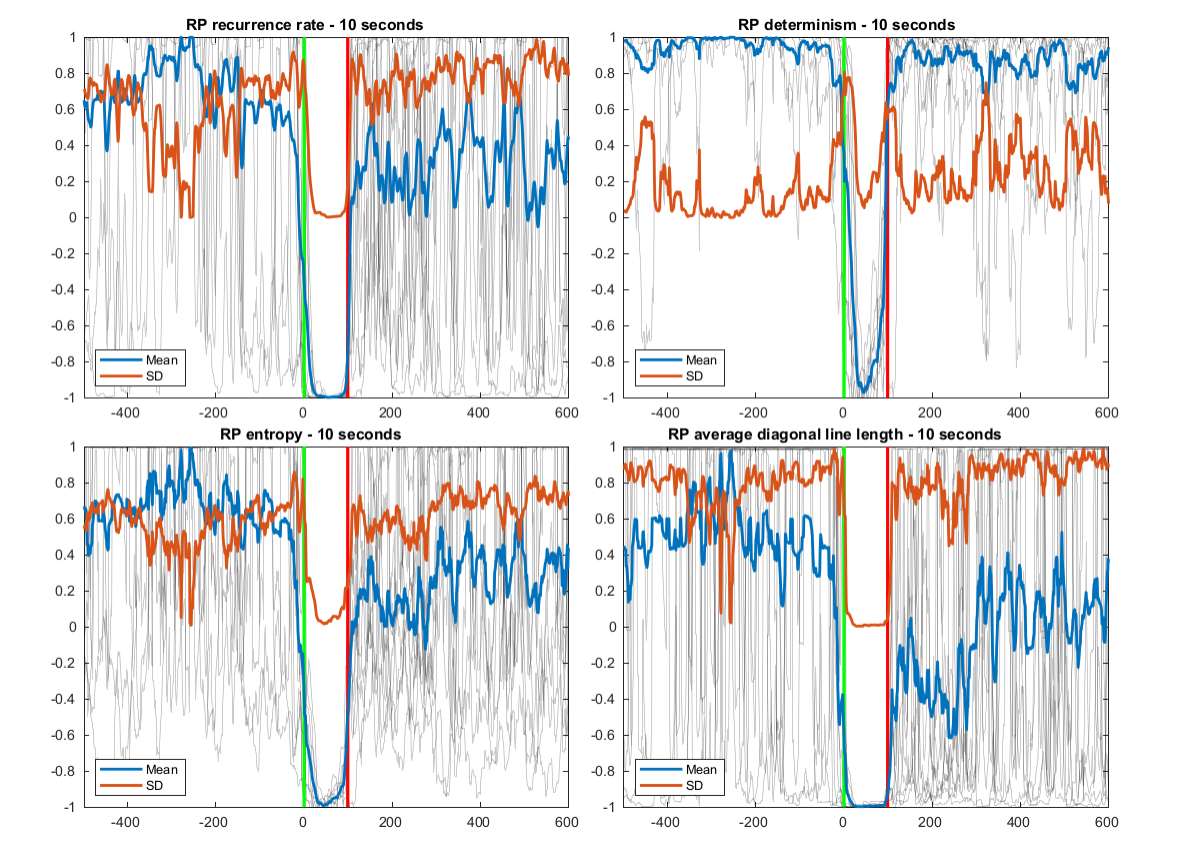
The overlaid feature value graphs for the recurrence plot features calculated from 10-second windows of the accelerometry data. Graphs representing feature values for each individual seizure (gray, background) are overlaid by the mean (blue) and SD (red). The green and red vertical bars represent the seizure onset and offset, respectively. The horizontal axis shows time in seconds related to seizure onset. All features are normalized between −1 and 1, independent from each other. RP: recurrence plot.

As detection models usually perform most effectively with smaller feature sets, both in terms of computational cost and prediction performance [31], we aimed to reduce the number of used features significantly. For this feature selection, we first looked at related literature in the field of wearable seizure detection to narrow down window sizes that effectively capture relevant signal changes in time and identify feature types that were successfully used previously. Therefore, we selected a window of 10 seconds for the ACC features [13,14,16] and a longer window of 5 minutes for the EDA features to capture the tonic changes in the EDA signal that evolve over longer periods [19]. We then visualized the feature data in a period around the seizure, overlaid over each other, and for all features separately. In addition, we plotted the mean and SD for each data series. The data that were used for these graphs were taken only from the seizures of participants that were not included in the test set to be used in the out-of-sample performance evaluation (see *Results* section). Features showing recurrent typical ictal changes were then visually selected for further analysis (Figure 1). Variable seizure durations were handled by upsampling shorter seizures by linear interpolation to the length of the longest seizure among those plotted.

The resulting feature subset for the ACC modality consisted of the magnitude, zero crossing rate, and recurrence plot features (Figure 1) [32]. For the EDA features, the area under the curve and the maximum of the SCL within the window, and the SCRR were chosen, all corrected against a baseline, which is an interval of the same duration as the feature window, ending immediately before the beginning of the feature window. Thus, the resulting feature set can be divided into 4 main feature groups:

1. Magnitude of the ACC signal 
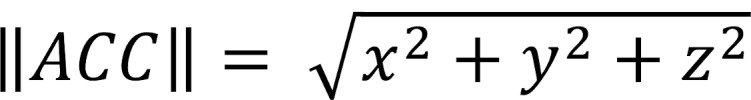
  - Raw ACC signal, over a 10-second window.
  - Zero-phase band pass filtered ACC signal over a 10-second window. The band pass filter had a frequency band of 0.1 Hz to 10 Hz, representing the linear component of the ACC signal, and was applied before segmentation into windows.
  - Zero-phase low-pass filtered ACC signal over a 10-second window. The low-pass filter had a cutoff frequency of 1 Hz, thus preserving only the gravitational component of the ACC signal, and was applied before segmentation into windows.
2. Zero crossing rate of the ACC signal over a 10-second window, for each of the 3 axes, respectively. The zero crossing rate is the number of times in a certain period the signal crosses the value 0 over the same period.
3. Four features calculated from the recurrence plot of the ACC signal:
  - Determinism, that is, the percentage of points that form diagonal lines of a minimal length.
  - The Shannon entropy of the probability that a line has a certain length.
  - The average diagonal line length.
  - Recurrence rate, that is, the density of recurrence points.
4. EDA-based features over a 5-minute window, minus the same value in the 5 minutes before the feature window
  - The area under the curve of the SCL was calculated as the moving mean of the raw EDA signal over a 1-minute window.
  - The maximum value of the SCL calculated as above.
  - The SCRR was calculated as the number of threshold crossings of the first derivative of the smoothed EDA signal within the window.

To accommodate the different window sizes over which the ACC and EDA features are calculated, a fixed interval between feature window applications was applied. This means that all features are calculated at fixed time points, with their respective windows centered on each consecutive point, creating the same number of feature vectors for both the ACC and EDA features over a segment of data. This enables the use of the complete, merged feature space as the single input into a detection model for training [11]. We chose this interval between the fixed time points for feature calculation as 2 seconds.

### Seizure Detection

We used a gradient tree boosting machine (GTBM) [33] as the detection model for TCSs. Although similar to the well-known random forest (RF) method in being a set of trees that are grown with training data, a GTBM builds trees as weak learners in an additive manner. The model is improved with each new weak learner that is added to the ensemble, whereas the RF model trains all trees in parallel and independent of each other. Weak learners in this case are trees with a very low number of splits, down to decision stumps with just 1 split. This results in an overall lower bias and similar variance for GTBM models compared with RF models at the cost of higher parameter tuning effort. Therefore, gradient tree boosting models generally perform better than RF models if tuned sufficiently, and they have been successfully used in many machine learning problems [34]. To tackle this tuning effort, we performed hyperparameter optimization over several of the model parameters in a leave-one-participant-out (LOPO) manner. To this end, the data set was split into a training set and a test set. The training set consisted of the 10-minute peri-ictal data of 10 TCS from 8 patients with epilepsy recruited at the Freiburg site. The basic test set consisted of the complete data from 2 patients, 1 from the Freiburg site and 1 from the London site with 11 TCSs (see *Results* section). The hyperparameter optimization only used the training set to keep the test set unknown to the model before testing. All feature data were normalized between −1 and 1 before training and testing. For training, the combined feature input for the model, that is, the peri-ictal feature data of 10 TCS, were normalized, and for testing the complete feature data from the recordings for a participant were normalized independent from the feature data of the other participants in the test set.

The hyperparameter optimization was performed in a LOPO nested cross-validation manner on the training set. The data for 1 of the 8 participants in the training set were kept back as a validation set, and the model was trained on the seizures from the other 7 participants, using only 10-minute peri-ictal data for each seizure. This reduction of the training data to only a small period around seizures helps with the large imbalance in the data set when comparing ictal and nonictal epochs. Once the model was trained, it was then tested on the complete data of the validation participant in the respective round, and the process was repeated 7 more times, cycling through the participants for validation. The mean score of the 8 validation runs was then saved as the performance of the current parameter combination, and the entire validation process was repeated for the next parameter combination. The parameters that were tuned in the optimization and their divisions are listed in Table 1, with the resulting optimal parameter combination highlighted. In total, 720 parameter combinations were evaluated in the hyperparameter optimization process.

Furthermore, the GTBM model also had some fixed parameters that were the same for all optimization runs. The boosting method used in the model was *adaptive boosting for binary classification* [35], and the misclassification cost for false negatives was always 1. The hyperparameter optimization resulted in an optimal set of parameters that were subsequently used in all the testing steps. The optimal parameter combination was chosen as the combination that achieved the highest sensitivity and lowest false alarm rate (FAR) during the LOPO validation run of the parameter combination, prioritizing sensitivity. Model parameters not specified here were left at their default values.

**Table 1:** Parameters optimized in the gradient tree boosting machine hyperparameter optimization and their optimization ranges.

Parameter	Value range	Description
Learning rate	1, *0.1*^a^, 0.01, 0.001	The step size in the iterative learning process, also called shrinkage
Number of trees	25, 50, 100, *250*, 500, 750	The maximum number of trees to produce in the model
False positive cost	1, 10, 20, 30, 40, *50*	Specific misclassification cost for false positives when weighting during the learning process
Tree depth	*1*, 2, 4, 8, −1	The maximum number of splits in the decision tree, where −1 denotes one less than the number of samples in the training set, that is, the maximum possible value

^a^The chosen optimal parameter combination are italicized.

### Evaluation

To process the model output and score its performance when compared with the ground truth, the same method was used both in the validation during hyperparameter optimization and later during the testing phase (see *Results* section). Owing to the method of feature extraction at fixed time intervals of 2 seconds described in the *Features* section, the output of the GTBM model is a prediction vector containing the predicted label every 2 seconds. The input labels, that is, the ground truth, and the predicted labels were binary, denoting the classification of each 2-second interval to either belong to a seizure or not. Comparing the ground truth and the prediction labels for evaluation can be done sample-wise by comparing each 2-second interval, or event-wise, by combining consecutive intervals of the positive class to distinct events. In our analysis, we chose the latter method, which requires postprocessing of the model output.

First, the prediction output of the model was smoothed with a hysteresis-like filter to avoid single-sample positives or gaps in consecutive positive predictions. To this end, all gaps between consecutive positive predictions smaller than 20 seconds in duration were filled out as positive, thus creating continuous, longer events from short neighboring positive predictions. Thereafter, all consecutive positive predictions of a certain length were discarded. We chose this value as 4 seconds, as it provides a good balance between discarding short, single-sample predictions and still keeping possible significant events. Thus, the prediction output of the model can be matched to the ground truth per participant by counting overlaps of predicted positive events with a positive ground truth event as true positives (TPs) and predicted positive events with no overlaps in the ground truth as false positives (FPs). The number of false negatives is then the difference between TPs and the number of seizures a participant recorded. The number of true negatives was not considered for this evaluation, as the sensitivity 
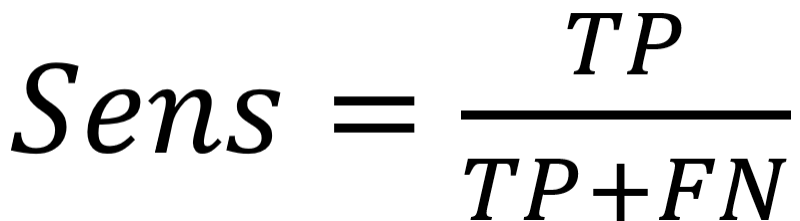
 and 
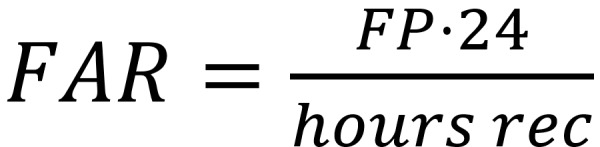
 are sufficient to evaluate a methodology for seizure detection. Unless otherwise stated, we report the sensitivity and FAR calculated across all relevant participants as a whole, not the mean over single participants.

All calculations for signal processing, feature extraction, and model development and evaluation were performed using *MATLAB 2020a* (MathWorks).

## Results

### Overview

For the study presented here, only study participants with focal to bilateral or generalized TCSs were included. This resulted in a data set of 21 TCSs from 10 participants, 9 from the Freiburg site with 19 seizures captured, and 1 from the London site with 2 seizures captured. The mean length of convulsive motor phenomena was 64 (SD 23) seconds. Table 2 lists the clinical and demographic information of the participants. They were 40% (4/10) female and on average 32.7 (SD 11.2) years old. The etiology of epilepsy for 2 participants was unknown at the time of recruitment. A total of 1 participant was diagnosed with generalized epilepsy, and the other 9 were diagnosed with focal epilepsy. For all captured seizures, wearable device data for at least 30 minutes before and after the ictal period were recorded in good quality; that is, the recorded data showed no major artifacts or intervals with constant 0 amplitude on visual inspection. A total of 612.6 hours of data were recorded for the included participants with seizures.

**Table 2:** Participants with recorded tonic-clonic seizure that were included in this study. Wearable data recorded from these participants were used in the evaluation of our seizure detection model. The recording duration is the duration that participants were wearing the device, without accounting for data loss.

Participant ID	Gender	Age (years)	Recording duration (days)	Epilepsy origin	Epilepsy type
FR1	Female	35	5	Unknown	Focal (TLE^a^)
FR2	Female	26	6	Structural	Focal (TLE)
FR3	Male	22	4	Genetic	Generalized (IGE^b^)
FR4	Female	34	4	Unknown	Focal (FLE^c^)
FR5	Male	56	8	Structural	Focal (TLE)
FR6	Male	38	7	Structural	Focal (TLE)
FR7	Male	25	4	Structural	Focal (xTLE^d^)
FR8	Male	16	7	Structural	Focal (FLE)
FR9	Male	37	12	Structural	Focal (xTLE)
LO1	Female	38	6	Structural	Focal (TLE)

^a^TLE: temporal lobe epilepsy.

^b^IGE: idiopathic generalized epilepsy.

^c^FLE: frontal lobe epilepsy.

^d^xTLE: extratemporal lobe epilepsy.

### Cross-validation Training

The training set used for hyperparameter optimization included 10 seizures from 8 participants and covered 414.7 hours of wearable device data. With the best parameter combination, as described above, the LOPO cross-validation could detect all 10 seizures (sensitivity=100%) with a total of 8 FPs (FAR 0.46 per 24 hours). The FP rate was calculated as the ratio of total FPs across all participants to the number of hours of recordings multiplied by 24, and not the mean FAR across participants. In the training set LOPO cross-validation, 75% (6/8) of FPs were produced from the data of 1 participant and 2 by another. Thus, the other 6 participants were free of FPs. All 8 FPs detected by the model during the LOPO cross-validation occurred when the patient was off camera, for example, in the morning or evening when they were in the bathroom for their daily washing routine.

### Out-of-Sample Testing

We also tested the model using a previously unseen test set from our overall data set. This test set included 11 seizures from 2 participants, 1 from the London site with 2 seizures recorded, and the other from the Freiburg site with 9 seizures recorded, for a total of 197.9 hours of test data. The choice of training and test set was deliberate: With the relatively low number of seizures and their distribution among participants in this data set, the goal was to train as many participants as possible but also having approximately the same number of seizures in the test set. This allocation ensures a model that is not patient specific while keeping the training and test sets balanced in terms of the number of seizures.

The GTBM model with the optimal parameters and trained with all 10 seizures from the training set could detect 10 of the 11 seizures in this test set (sensitivity=91%), without any FPs. However, this test set was rather limited as it was biased toward participants who had convulsive seizures; therefore, we expanded the test set to also include data from all 30 patients with epilepsy recruited at the London site that had data recorded with the wearable device. Although this does not add more seizures for the model to detect, it does add a considerable amount of data to assess the FP rate. The expanded test set thus encompasses 1935.9 hours of wearable device data from 31 participants, including the same 11 seizures as before. In this data set, the same model produces 30 FPs (0.37 per 24 hours). Further investigation of the FP distribution among the participants showed that 15 false detections resulted from a single participant who used a stepper during monitoring as physical activity to trigger her seizures. All FPs for that participant were related to this activity. Removing this participant performing unnatural repetitive movements from the expanded test set lowers the FP rate to 0.19 per 24 hours. Of the other participants in this expanded test set, the data of 2 participants produced 3 FPs, respectively, whereas 9 other participants each produced 1 FP, with the remaining 19 participants being free of FPs. Thus, the FAR, when calculated as the mean across all the included participants’ individual FARs, was 0.45 (SD 1.1) per 24 hours, and 0.29 (SD 0.53) per 24 hours when excluding the participant with 15 FP. Table 3 provides a detailed overview of the results among the participants with recorded seizures.

**Table 3:** Per participant evaluation results, for participants with seizures recorded. The 3 totals given for the test set are (1) the total across the test set participants with seizures recorded (N=2), (2) the total when including all patients with epilepsy recruited at the London site with data recorded (not listed, N=31), and (3) the total when excluding 1 participant with an artificially disproportionate number of false positives (N=30).

Participant ID			Sensitivity, n (%)		FP^a^, n		FAR^b^ (per 24 hours)		PPV^c^ (%)		Recording length (hours), n		Seizure type
**Training set**													
	FR1	1 (100)		0		0		100		59.6		sGTCS^d^	
	FR2	1 (100)		6		1.56		14		92		sGTCS	
	FR3	2 (100)		0		0		100		35.5		GTCS^e^	
	FR4	1 (100)		2		1.34		33		35.8		sGTCS	
	FR5	1 (100)		0		0		100		36.3		sGTCS	
	FR6	1 (100)		0		0		100		88.5		sGTCS	
	FR7	1 (100)		0		0		100		40.7		sGTCS	
	FR8	2 (100)		0		0		100		26.2		sGTCS	
	Total	10 (100)		8		0.46		56		414.7		N/A^f^	
**Test set**													
	FR9	9 (100)		0		0		100		112.2		sGTCS	
	LO1	1 (50)		0		0		100		85.7		sGTCS	
	Total (1)	10 (91)		0		0		100		197.9		N/A	
	Total (2)	10 (91)		30		0.37		25		1935.9		N/A	
	Total (3)	10 (91)		15		0.19		40		1870.3		N/A	

^a^FP: false positive.

^b^FAR: false alarm rate.

^c^PPV: positive predictive value.

^d^sGTCS: focal to bilateral tonic-clonic seizure.

^e^GTCS: generalized tonic-clonic seizure.

^f^N/A: not applicable.

### Seizure Duration

**Figure 2:**
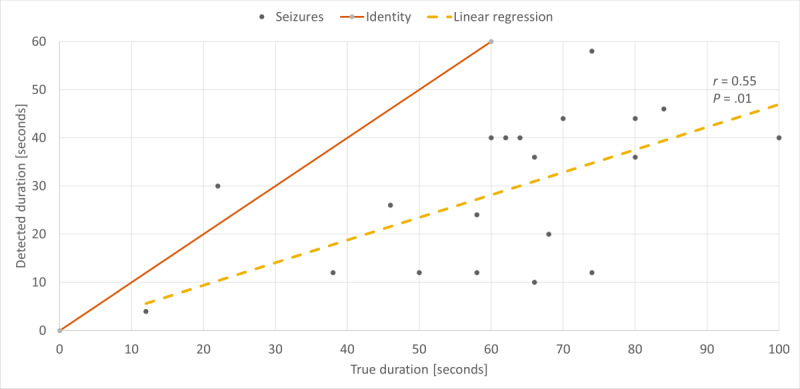
Correlation of the true seizure durations as labeled by clinical experts and the ictal durations detected by the gradient tree boosting machine model based on accelerometry and electrodermal activity. The dotted line shows the linear regression fit across the data points. The Pearson correlation coefficient was r=0.55, with *P*=.01. The identity line shows that the seizure duration is generally underestimated by the model.

The duration of detected seizures was significantly correlated with the video EEG–based seizure duration, as labeled by clinical experts (Figure 2). The true seizure duration here is based on its clinical manifestation, that is, onset until offset of ictal motor phenomena related to TCS. In a Pearson correlation test, the correlation coefficient was *r*=.55, with *P*=.01. In general, the seizure duration was underestimated by the model as approximately half of the true duration, with a mean identified duration of 29 (SD 15) seconds versus the mean seizure duration of 64 (SD 23) seconds. This may reflect minor movement amplitudes during the tonic phase of the TCSs.

### Feature Importance

**Figure 3:**
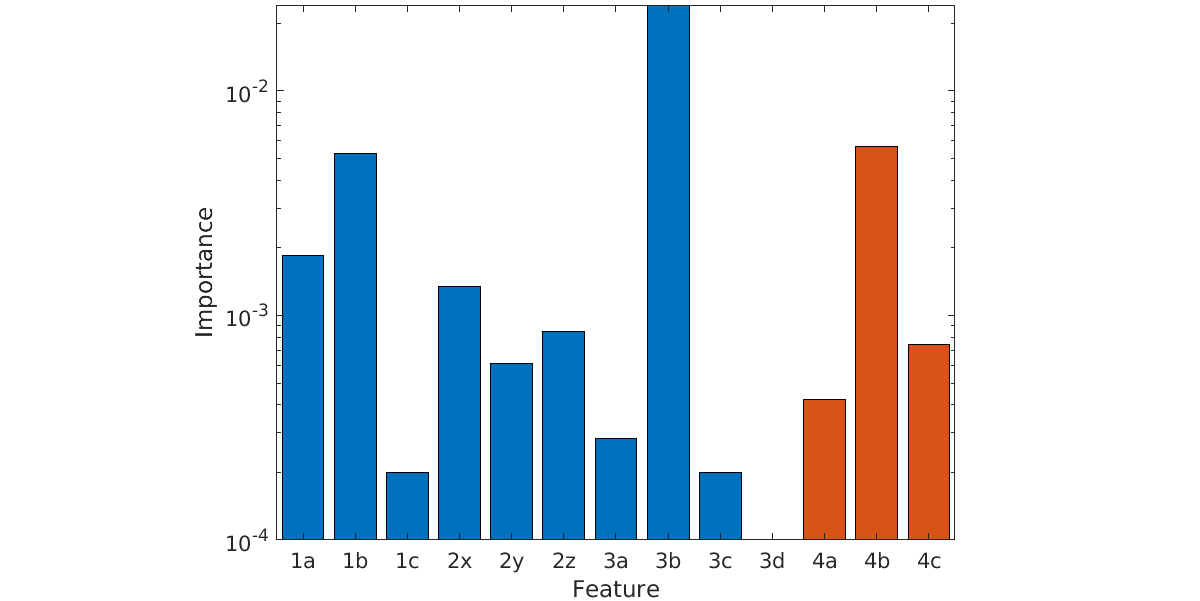
Feature importance, calculated as the mean feature importance of all models during a leave-one-participant-out cross-validation, with the optimal parameters of the gradient tree boosting machine as reported in the Seizure Detection section. All the features are shown as listed in the Features section (1: magnitude of accelerometry, 2: zero crossing rate of accelerometry, 3: recurrence plot features of accelerometry, and 4: electrodermal activity features). The feature importance is shown in logarithmic scale to better visualize smaller differences.

Furthermore, we analyzed the feature importance for our feature set, calculated as the mean feature importance over all trained GTBM models in the LOPO cross-validation (Figure 3), as a metric for the contribution of a specific feature to the performance of the model. The feature importance was based on the Gini impurity, calculated such that the smallest possible value was 0 [36]. Overall, all 4 feature groups, as described in the *Features* section, are represented in the resulting GTBM model to varying degrees of importance. The top 3 features among the feature set were that the Shannon entropy of the probability that a line in the recurrence plot had a certain length calculated over a 10-second window of the ACC signal, the magnitude of the band pass filtered ACC signal in a 10-second window, and the maximum of the SCL in a 5-minute window of the EDA signal, corrected for a baseline.

## Discussion

### Principal Findings

The results show that the GTBM model can robustly detect TCSs from non–EEG wearable device data. A sensitivity of 100% (10/10) on the training set during a LOPO cross-validation, a sensitivity of 91% (10/11) on the out-of-sample test set, and an FAR of less than 1 per 5 days in more than 1800 hours of data indicates a sufficient robustness of this methodology to consider it in designing an automated seizure diary. A large percentage of FPs occurred in a small percentage of participants, with most other participants showing between 0 and 0.5 FP per day. Furthermore, in participants who had TCS in our test set, no FPs were reported by the model. In addition, all true detections of our model occurred within the ictal period of the respective seizure, showing that the system has high accuracy. By evaluating a test set that includes data largely from 1 site (London), while the model was trained exclusively with data from the other site (Freiburg), we also showed the generalizability of our model.

Although our data set contains continuous circadian data, most TCSs occurred during nighttime sleep. In the training set, 50% (5/10) of seizures occurred while the patient was awake, and in the test set, only 9% (1/11) occurred during wakefulness. Of these 6 awake seizures, 2 seizures occurred when the patient was outside the bed. All TP detections, both in the training set LOPO cross-validation and in the test set evaluation, occurred within the ictal phase of the respective seizure. Conversely, all FP detections occurred when the patient was awake and active, and most of them occurred during daytime. Patients were generally not confined to their beds but rather to their hospital rooms. They could freely perform a variety of activities of daily living, such as strolling across the room, going to the bathroom, brushing their teeth, eating and drinking, and washing themselves. Movement patterns during these activities, particularly if repetitive, could resemble those during convulsive seizures and may be a common source of FP detections. However, false alarms during these activities when the patient is awake could be ignored easily by way of patient validation and feedback to avoid inappropriate interventions.

### Feature Importance

The distribution of feature contribution to the performance of the model shows that all selected features are used by the model to predict a seizure event, except for one, the recurrence rate in the recurrence plot of the ACC signal. The least amount of importance is assigned to the magnitude of the low-pass filtered ACC signal. This is an expected outcome, as this feature represents the gravitational component of the movement, which is minimal during convulsive seizures. During these seizures, almost all movements are part of the linear component, represented by the band pass filtered signal, which is also confirmed by this feature being one of the most important in the model.

**Figure 4:**
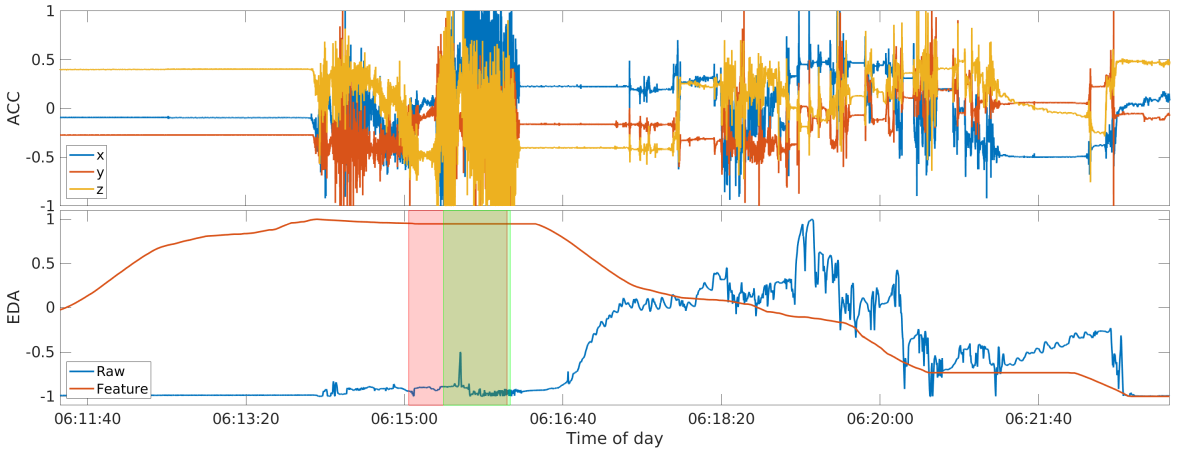
The seizure of participant LO1 that was detected by the model. The raw accelerometry signal is shown at the top, and the raw electrodermal activity signal as well as the best electrodermal activity feature (Section Features, Feature 4b) at the bottom; all are normalized between −1 and 1, independent from each other. The ictal tonic-clonic phase is overlaid in red, the true positive detection is overlaid in green. ACC: accelerometry; EDA: electrodermal activity.

Among the EDA-derived features, the highest importance was consistently assigned to the difference between the highest value in the feature and the baseline windows of the SCL. A typical EDA signal progression in the peri-ictal period is a steep increase from a low preictal baseline during the ictal phase, followed by a shallow decrease in the postictal phase, spanning multiple minutes. Thus, the feature based on the difference of the highest value between preictal, ictal, and postictal phases can sufficiently represent this trend, as evidenced by its high importance. Figure 4 shows the EDA signal progression and the respective maximum SCL feature during a seizure. The feature values are at their highest during the ictal phase, whereas the raw EDA signal shows the typical progression described above.

### False Negatives

**Figure 5:**
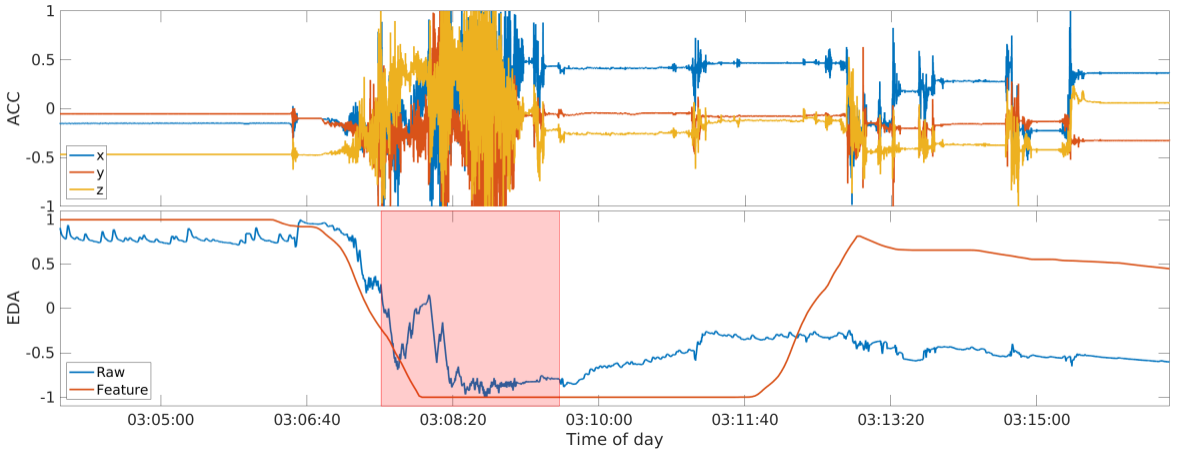
The seizure of participant LO1 that was not detected by the model and the single false negative that was produced during the evaluation. Note the differences in the electrodermal activity signal progression in comparison to Figure 4, which shows a typical response. The raw accelerometry signal is shown at the top, and the raw electrodermal activity signal and the best electrodermal activity feature (Section Features, Feature 4b) at the bottom; all are normalized between −1 and 1, independent from each other. The ictal tonic-clonic phase is overlaid in red. ACC: accelerometry; EDA: electrodermal activity.

There was 1 seizure the model did not detect among the training and testing data sets (Figure 5). This false negative was produced by one of the participants recruited at the London site, and the seizure occurred during the night when the patient was asleep. The other seizure recorded for this participant was successfully detected by the model. To explain why the seizure was rejected by the model, we examined the raw data before and after the seizure, specifically looking at the ACC response during the seizure, and the EDA trend going from the pre- to postictal phase. The motion response in the ictal phase of the rejected seizure was a typical progression from a short tonic phase at the beginning of the seizure to a longer, very pronounced, and violent clonic phase, stopping promptly with the seizure offset, followed by a short phase of postictal ACC silence. The raw EDA signal, however, follows a progression directly opposite to the signals from all other TCSs in the data set. The signal shows a steep decrease from a high baseline during the ictal phase and remains at a lower level in the postictal phase compared with the baseline in the preictal phase. Figures 4 and 5 show the comparison of data from the 2 recorded seizures from participant LO1, with the detected seizure being representative of all other TCS in the data set, especially those in the training set that created the model. Both seizures showed similar ACC data and a similar change in the ACC-based feature values. However, the EDA data and feature values were visibly opposite. This confirms that the model was trained properly on both the ACC and EDA features and that both modalities contributed to the model’s classification of seizure occurrence. Thus, the misclassification of 1 event was due to atypical raw data and confirmed that the model included EDA features in its classification.

A possible explanation for the unusual EDA signal during this seizure could be that the EDA electrodes lost adequate contact with the skin, which was not fully re-established after the seizure. This could be caused by an improperly worn wearable device, or a loss of contact owing to the wearable device coming into contact with an external obstacle such as being pressed into the bed, slightly raising the EDA electrodes off the skin.

### Related Work

The research that is most closely related to our premise is certainly that of Onorati et al [16]. In their work, the Empatica research group developed a seizure detection model based on wearable data from the same device used in this study, *Empatica E4*. They used a support vector machine trained with 25 ACC as well as EDA features that were not further specified to detect convulsive seizures and achieve a very good performance, with their best classifier reaching a sensitivity of 94.5% and an FAR of 0.2 per day on 55 seizures from 22 patients. Our approach is on par with their results, and a contribution of the work presented here is to reinforce their findings. We show that the results of this quality can be achieved with a relatively basic methodology, and we describe this methodology in greater detail, making it fully accessible and reproducible. The methodology may even be transferrable to other diseases with convulsive attacks, such as paroxysmal dystonia or dissociative seizures. Thus, the study described here could be used as a stepping-stone for further work not only in epilepsy research but also in other medical fields.

In a further study, Kusmakar et al [13] used a monomodal support vector data description model on wearable ACC data to detect 21 generalized TCS from 12 patients, with a total recording length of 966 hours. The outlier classification model could achieve a sensitivity of 95% in a LOPO cross-validation, with a mean FAR of 0.72 per day. However, their model generated FP detections across almost all of the 12 included patients, showing a general trend toward FP detections independent of patient selection, whereas our model could achieve a generally lower FP rate on both the training and test sets, also revealing certain patients with a disproportionate FAR.

Arends et al [17] used the *LivAssured NightWatch* wearable device in a large ambulatory long-term monitoring study, collecting 908 convulsive seizures from 28 patients over more than 1800 nights. The device collects ACC and PPG signals from the patients’ upper arm, specifically during the night. Their thresholding algorithm could detect 86% of the recorded seizures, with a positive predictive value of 49%, indicating that roughly half of all predictions were FPs. Although our methodology produces slightly worse results with respect to the overall FAR, studies differ in that the *NightWatch* study only assessed nocturnal data with patients at rest, whereas our assessment, based on continuous data comprising wakefulness and sleep, showed the model’s ability to correctly detect daytime seizures; notably, all our FPs were generated while the respective patient was awake and active.

In a more recent study, Johansson et al [14] used wrist-worn ACC sensors to detect 37 TCS from 11 patients with 666 hours of data. They evaluated 3 different types of models on a test set of 10 seizures and obtained the best result using an RF algorithm, detecting 9 of 10 seizures with an FAR of 0.24 per day. However, the evaluation of FPs is constrained in patients with TCS, introducing a certain bias in patient selection. In our evaluation, we added a control group of up to 29 participants without TCS recorded, with our model achieving a similar FAR, while also only producing FPs on these participants without seizures, whereas the participants with TCSs had no false alarms.

### Limitations

The methodology for TCS detection described here also introduces some limitations, one of which is the long feature window used for the EDA feature computation. To include tonic changes in the EDA spanning over multiple minutes in the postictal phase, we used a 5-minute-long window, which automatically introduces an inherent detection delay, as a real-time system would need to first collect these data before being able to extract the EDA features and detect a potential seizure. Thus, this methodology would not be suited as a real-time warning system. Another limitation is the constraint of the model to detect only TCSs. As the model training process relies on data from the accelerometer sensor, nonmotor seizures cannot be detected with this set of modalities and features. Future work will be needed to assess the contribution of PPG and EDA sensors in detecting nonmotor seizures. Furthermore, the performance of the specific model trained here is likely not sufficient to be deployed directly as an automatic seizure diary, especially considering its constraint on TCS, which can be infrequent in everyday life. Additional work and more training data would be needed to create a system that is usable in clinical practice, possibly even shifting to a semipersonalized model that can be reinforced over time by patient feedback.

One of the most prevalent limitations in many studies in this field is the controlled in-hospital setting in which wearable device data are collected. Although patients in our study were able to perform some activities of daily living in and around their bed and were able to walk within their hospital room, the likelihood of FP generation can be assumed to be higher in an outpatient setting. False alarms during physical activity could be addressed by actively involving the patient through validation and feedback, for example, by giving them a chance to review seizure diary entries. Nevertheless, transferring this methodology to an ambulatory setting will require extensive modifications and reevaluation with data recorded in everyday living situations that include a gold standard for seizure labeling. In any case, a robust classifier that has a likelihood of working in the field must first be validated in an inpatient setting to progress to an ambulatory study, and the research presented here takes a clear step in that direction.

## Abbreviations

ACC: accelerometry
ECG: electrocardiography
EDA: electrodermal activity
EEG: electroencephalography
EMU: epilepsy monitoring unit
FAR: false alarm rate
FP: false positive
GTBM: gradient tree boosting machine
LOPO: leave-one-participant-out
Non-EEG: nonelectroencephalography
PPG: photoplethysmography
RF: random forest
SCL: skin conductance level
SCRR: skin conductance response rate
TCS: tonic-clonic seizure
TP: true positive
